# Clinician perspectives on blood culture practice and diagnostic stewardship in regional emergency departments

**DOI:** 10.1099/jmm.0.002171

**Published:** 2026-06-11

**Authors:** Elizabeth Thomas, Monalyssa Watson, Tim Inglis, Stephen MacDonald

**Affiliations:** 1 School of Population Health, Curtin University, Perth, Australia; 2 Medical School, The University of Western Australia, Perth, Australia; 3 Western Australian Country Health Services, Perth, Australia; 4 Pathwest, Perth, Australia

**Keywords:** antimicrobial stewardship, blood culture, clinician perspectives, diagnostic stewardship, emergency department, qualitative research, rural health, sepsis, Western Australia

## Abstract

**Introduction.** Bloodstream infection is a critical cause of morbidity and mortality, and high-quality blood culture practice is essential for accurate diagnosis and antimicrobial stewardship, particularly in regional and remote healthcare settings.

**Gap Statement.** Clinicians working in rural emergency departments (EDs) encounter unique systemic, workforce and logistical barriers to optimal blood culture practice, yet these challenges are not well characterized in the existing literature.

**Aim.** To explore clinician-reported barriers and enablers to high-quality blood culture collection across regional EDs in Western Australia.

**Methodology.** A qualitative study was conducted involving semi-structured interviews with doctors and nurses from three Western Australia Country Health Service EDs. Transcripts were analysed thematically to identify factors influencing blood culture ordering, collection technique, contamination and workflow integration.

**Results.** Twenty-four clinicians participated, describing substantial delays between sample collection and laboratory processing, difficulty obtaining two sets per episode and limited feedback on contamination or blood volume adequacy. Workforce turnover, variable training and inconsistent guideline use contributed to practice variation. Key enablers included strong team culture, leadership from clinical champions, structured induction, refresher training and the use of dual-set kits and visual dashboards to provide timely, non-punitive feedback.

**Conclusion.** High-quality blood culture practice in rural EDs is shaped by multifactorial systemic, workflow and workforce constraints and targeted, locally relevant interventions are essential to improve diagnostic yield and strengthen antimicrobial stewardship.

**Impact Statement**

This study provides the first in-depth qualitative examination of blood culture practices in regional Australian emergency departments, revealing how systemic, workflow and workforce constraints shape diagnostic quality. By identifying practical, context-specific enablers, the findings offer a clear pathway for improving diagnostic stewardship and supporting safer, more effective sepsis care in rural and remote settings.

## Data summary

No external datasets, software or code were generated or reused in this study. The research was based entirely on qualitative interview data that are not publicly available due to confidentiality requirements under the approved human research ethics protocols. Additional data cannot be shared publicly but may be made available on reasonable request to the corresponding author, subject to ethics approval.

## Introduction

Sepsis is a major global health challenge, responsible for an estimated 49 million cases and 11 million deaths annually, accounting for almost one in five deaths worldwide [1]. In Australia, sepsis is estimated to cause more than 100,000 hospitalizations each year, with mortality approaching 10% and disproportionately higher rates in rural and remote regions [2,3]. Survivors frequently experience long-term disability, and the associated health system burden is substantial due to prolonged hospitalization and intensive care requirements [4,5]. Timely diagnosis and effective treatment are essential for improving outcomes [6]. The detection of pathogenic organisms in blood is required to confirm bloodstream infection and direct target antimicrobial therapy [6].

Blood cultures are the reference standard for detecting bloodstream infection [7]. High-quality blood culture collection, including aseptic collection technique, adequate blood volume and pre-antibiotic sampling, optimize diagnostic yield and reduces contamination [7,8]. However, these processes are inconsistently followed, leading to delayed or false-negative results [9,10]. In regional healthcare settings, the additional delay between sample collection and receipt in the laboratory can significantly extend the overall time to a result [11]. This leads to prolonged empiric broad-spectrum antibiotic therapy, which contributes to antimicrobial overuse, resistance and unnecessary patient exposure to adverse drug effects [12,14].

Studies from both high-income and low- and middle-income countries have highlighted challenges in recognizing sepsis and strengthening diagnostic capacity, particularly in settings with limited resources or workforce constraints [13,15, 16]. For example, qualitative research has shown that frontline clinicians often lack consistent training in blood culture best practice, leading to variation in collection volumes, technique and decision-making about when or whether to order tests [17,18]. Workforce shortages, particularly of experienced nursing staff and phlebotomists, have also been linked to higher contamination rates and missed opportunities for pre-antibiotic sampling [19,21]. These challenges compromize the value of blood cultures as a diagnostic tool and undermine antimicrobial stewardship efforts.

Contamination is a recurring concern in blood culture practice, with studies reporting rates of up to almost 8% in emergency departments (EDs) in high-income countries [22,24]. False-positive results can lead to unnecessary antimicrobial therapy, prolonged hospital stays and significant costs to the health system [12,20, 21]. Each contaminated culture or false positive result is estimated to add thousands of dollars to healthcare expenditure, in addition to the clinical consequences for patients [25]. Conversely, inadequate collection volumes reduce diagnostic sensitivity, leading to delay to detecting organisms where present or even missing them altogether [10,15]. Both issues underscore the importance of training, workflow optimization and feedback mechanisms to support high-quality blood culture practice.

Evidence suggests that targeted interventions can improve blood culture collection and utility [26]. For instance, audit and feedback systems that provide clinicians with contamination rates and volume compliance data have been shown to reduce errors and support behaviour change. Similarly, workflow redesign – such as provision of dual-set kits, standardization of equipment and embedding collection prompts into electronic order systems – can reduce variability and reinforce best practice. Leadership from local champions and a culture of accountability have also been recognized as powerful enablers, particularly in settings with frequent staff turnover. Despite these insights, little is known about how such barriers and enablers operate in rural and remote EDs, where structural challenges are more pronounced.

In Western Australia, the Western Australia Country Health Service (WACHS) provides care to a population of 566,731 across an area of more than 2.5 million square kilometres, encompassing some of the most remote regions in the world [27]. Challenges include lengthy transport distances to central laboratories, frequent employment of short-term locum and agency staff, and diverse patient populations spanning urban, rural, remote and Aboriginal communities [28]. Improving diagnostic capacity in such environments is a recognized priority for both patient safety and antimicrobial stewardship [15]. The Adaptive Diagnostics for Emerging Pathogenic Threats (ADEPT) project was established to address the challenge of emerging antimicrobial-resistant sepsis in regional and remote Australia by developing and testing strategies to strengthen diagnostic capability.

Within ADEPT, one critical area of focus is blood culture practice in EDs, where 81% of regional blood culture requests originate. While quantitative studies have described delays and contamination rates [14,16], there is limited qualitative evidence exploring how clinicians experience and navigate these challenges in practice. Understanding doctors' and nurses' perspectives is essential for designing feasible, context-specific interventions that improve blood culture quality and impact patient outcomes.

The present study, therefore, sought to explore the barriers and enablers to blood culture testing across three regional EDs in rural Western Australia. Semi-structured interviews and thematic analysis were employed to examine the systemic, workforce and cultural factors influencing practice and to identify opportunities for improvement aligned with local realities. By generating these insights, the study contributes to the growing evidence base for strengthening sepsis diagnosis and antimicrobial stewardship in rural and remote healthcare settings.

## Methods

### Study design and setting

This study used a qualitative design to explore clinician perspectives on blood culture practice in regional EDs to determine contextual, organizational and behavioural factors influencing diagnostic practice in rural and remote settings. Quantitative indicators such as contamination rates or turnaround times cannot fully explain the systemic, workflow and cultural influences that shape practice. Semi-structured interviews captured frontline clinicians’ experiences, perceptions and recommendations in their own words. The study was undertaken in three EDs within the WACHS. The sites were selected to represent a mix of regional and remote contexts, service sizes and workforce structures.

### Participants and recruitment

Participants were doctors and nurses currently practising in the EDs at the three study sites. Eligible clinicians included permanent and short-term staff with direct responsibility for patient assessment and management. Site coordinators distributed invitations via staff meetings and internal communications, and participation was voluntary. Interested staff contacted the research team directly to arrange an interview. All participants provided written informed consent before data collection, and data were stored in accordance with institutional policies on confidentiality and security.

### Data collection

Semi-structured interviews were conducted between April and June 2025, either face-to-face at the study site or via secure videoconferencing. Interviews followed a topic guide covering awareness and use of blood cultures, perceived barriers and enablers to appropriate testing, experiences of laboratory turnaround times, contamination and volume adequacy and views on potential interventions, such as feedback systems, training and workflow redesign. Interviews lasted 30–60 min and were audio-recorded with permission. Recordings were professionally transcribed verbatim and de-identified.

### Data analysis

Transcripts were analysed thematically using an inductive approach. Two researchers independently coded a subset of transcripts to develop a preliminary coding framework, which was then refined through discussion and applied to the whole dataset using NVivo software (QSR International). Codes were grouped into broader categories, and themes were derived iteratively by comparing patterns across participants and sites. Discrepancies were resolved through discussion until consensus was achieved. Themes were reviewed in relation to the research objectives and triangulated against field notes to enhance credibility.

## Results and discussion

### Participant characteristics

In total, 24 clinicians participated in the study, comprising 16 doctors (senior doctors – specialists, regional general practitioners – registrars and junior medical officers) and eight nurses. Participants represented a mix of permanent and short-term staff, reflecting the range of seniority and experience of the workforce in regional and remote EDs. This breadth of roles allowed for comparison of perspectives across professional groups and provided insights into both medical decision-making and practical collection processes. The inclusion of multiple sites ensured representation of different workforce stability profiles, from relatively stable regional centres to remote hospitals that rely heavily on short-term staff.

### Clinician recognition of the importance of blood cultures

Across all sites, participants consistently acknowledged the central role of blood cultures in diagnosing bloodstream infections and guiding sepsis management. Clinicians described blood cultures as ‘essential’ for confirming infection and tailoring therapy, aligning with international sepsis guidelines that emphasize early blood culture collection before antimicrobial administration. However, participants also noted that, in practice, blood cultures were frequently deprioritized during initial patient assessment, when immediate resuscitation and empiric therapy took precedence. This tension between guideline ideals and emergency realities reflects prior studies showing that, in real-world ED settings, fewer than half of eligible sepsis patients have cultures drawn before antibiotics.

### Workflow and systemic barriers

A recurring theme was the impact of systemic and logistical barriers on blood culture collection. Blood cultures collected in smaller hospitals require to be transported to a regional laboratory for processing. Delays in the commencement of incubation affect sensitivity and can lead to extended times to the final result of 100 h or more in some cases. These findings mirror international evidence that centralized laboratory models, while cost-efficient, contribute to diagnostic delays in rural and remote health systems. Participants also highlighted practical barriers within ED workflows, including competing demands during peak presentations and the absence of designated phlebotomy staff responsible for culture collection. Some staff described missed opportunities where cultures were ordered but not collected before antibiotics were given. Similar issues have been documented in other Australian and international studies, where workflow interruptions and unclear role delineation contribute to practice variation. Finally, the requirement to collect a second set of blood cultures from a separate phlebotomy site is recognized as a significant obstacle to compliance. Emerging evidence supports the collection of two sets from a single sterile venepuncture [29].

### Workforce challenges and turnover

Staff turnover, particularly reliance on short-term staff, was described as a barrier to consistent practice. New or transient staff often lack familiarity with local protocols or training in aseptic technique and optimal blood culture volume. This contributed to both under-collection and increased contamination risk. Workforce instability is a well-recognized issue in rural health services globally. Our findings demonstrate its potential downstream impact on diagnostic quality. Participants emphasized the need for structured induction and refresher training for all staff, echoing prior research showing that regular education programmes can reduce contamination rates and improve adherence to best practice.

### Contamination and volume adequacy

Several clinicians expressed concern about the lack of routine feedback on contamination or adequacy of blood culture volumes. Without such data, participants felt unable to assess their own performance or identify areas for improvement. This finding is consistent with evidence that audit and feedback are among the most effective quality improvement strategies for blood culture practice [30]. Contamination rates in Australian EDs are typically reported at 2–3%, but can exceed 5% in settings with high turnover and limited phlebotomy support [31,32]. Participants recognized that contamination is a problem, for example, prolonged or unnecessary antibiotic use triggered by false-positive results. The extent of the problem was unknown. Clinicians reported that the availability of data on contamination rates would motivate best practice and enhance confidence in the test’s value.

### Awareness and use of guidelines

There was a general consensus that blood cultures are collected in the ‘sickest’ patients, although there was variation in how this was defined. There was minimal knowledge of any decision rules (e.g. Shapiro [33]) to guide when blood cultures are indicated. Participants were generally aware of sepsis recognition pathways and national antimicrobial stewardship initiatives, but many reported that blood culture practice was less explicitly embedded into routine clinical pathways. Junior medical and nursing staff were more likely to follow such documents than older doctors, who described relying more on gestalt. Some clinicians noted that while sepsis bundles focus on the timeliness of antibiotic administration, there was no routine measure for monitoring blood cultures. Several participants expressed concern that the guideline’s emphasis on rapid antimicrobial administration may discourage staff from optimal collection of blood cultures beforehand, particularly a second set. This highlights the importance of balancing competing priorities in sepsis care and suggests that integration of blood culture collection into sepsis bundles should be strengthened.

### Perceptions of diagnostic value

Clinicians held mixed views on the diagnostic utility of blood cultures in the rural ED context. All recognized that blood cultures are not relevant for immediate management, noting that culture results occasionally identified unexpected organisms that altered management. Others, however, expressed scepticism about their usefulness given long turnaround times and low perceived yield, particularly in less unwell patients. This ambivalence mirrors studies showing variable clinician confidence in the clinical relevance of blood culture results, especially when empirical therapy already addresses likely pathogens. Importantly, such perceptions may influence ordering behaviour, with some staff admitting to selective or inconsistent use of cultures in patients with suspected sepsis. Addressing these attitudes through education and feedback could reinforce the clinical importance of high-quality cultures.

### Impact on patient flow and safety

Several participants noted that when patients require urgent transfer to a tertiary centre, it is common practice to send the blood culture with the patient to ensure prompt processing upon arrival. This approach reflects the adaptive strategies used in regional sites to maintain diagnostic continuity. Others reported that difficulties accessing equipment or trained staff could lead to missed or delayed collections, with potential implications for diagnostic accuracy and antibiotic stewardship. Several participants commented on the global shortage of blood culture bottles in 2024 [34], resulting in a degree of rationing of their use to the most unwell patients. These findings underscore the need to view blood culture practice as part of broader patient flow processes in emergency care. Literature from other acute care settings suggests that embedding diagnostic steps into streamlined workflows improves compliance and reduces missed opportunities [35,36].

### Cultural and relational dimensions

Beyond technical and logistical issues, participants emphasized the importance of team culture and relationships in shaping practice. Sites with strong interprofessional trust described smoother delegation of culture collection, while in other settings, hierarchical barriers sometimes delayed or prevented nurses from initiating collections. This variation reflects evidence that team dynamics influence adherence to sepsis protocols and diagnostic stewardship [37]. Promoting nurse-led models, with clear protocols and leadership support, helps overcome hierarchical constraints and reduce variability [38]. Clinical focus and leadership from hospital managers were recognized as a key driver of quality performance.

### Enablers: leadership, culture and champions

**Fig. 1.**
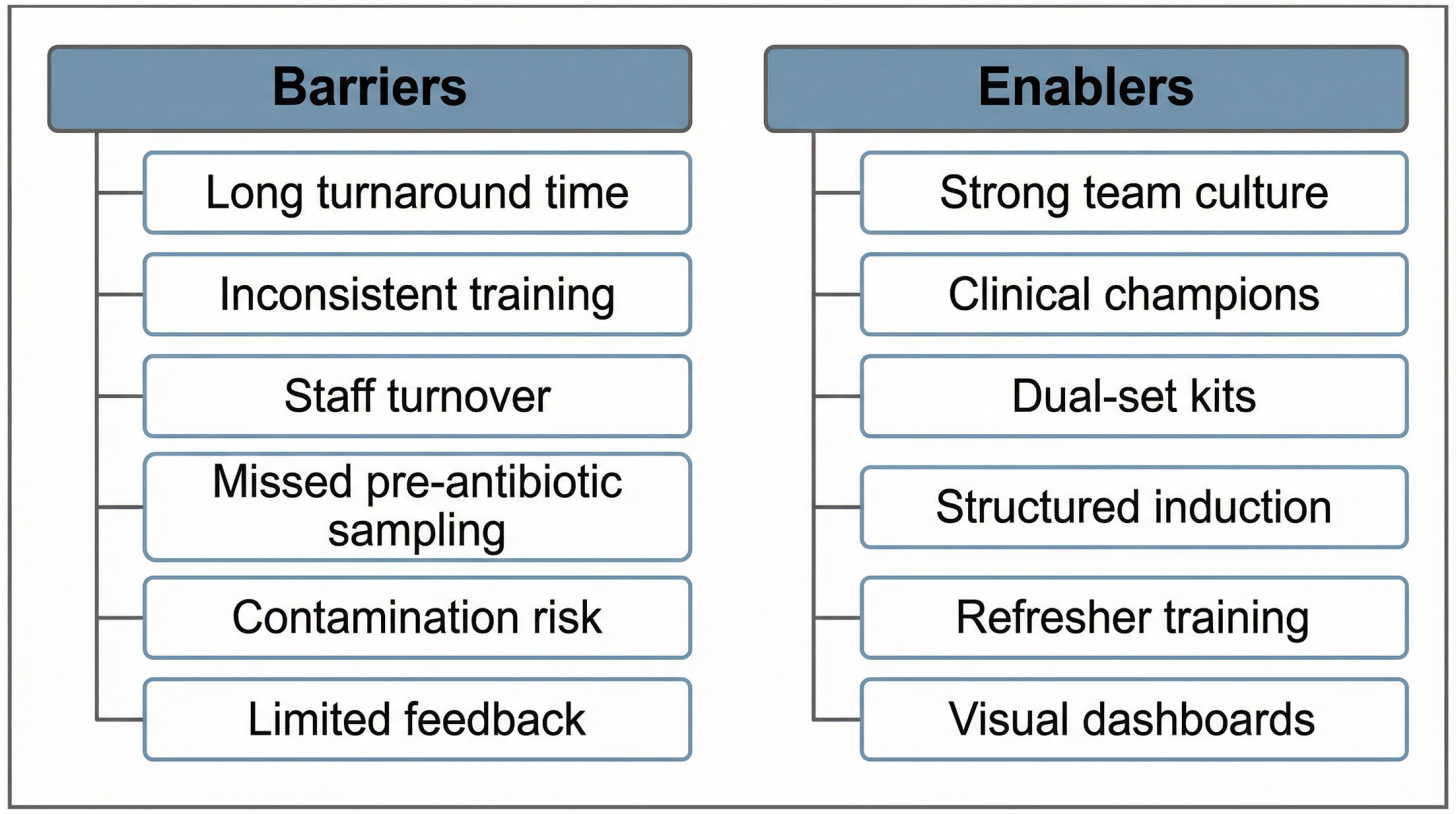
Barriers and enablers to optimal blood culture specimen collection.

Despite these barriers, participants identified several strong enablers (Fig. 1). Local clinical champions, often senior nurses or doctors, played a key role in modelling best practice, reinforcing standards and creating a culture of accountability. Team-based approaches, where nurses initiated cultures without waiting for medical orders, were viewed positively and helped reduce missed pre-antibiotic opportunities. Such findings resonate with previous studies showing that local leadership and strong team culture can mitigate systemic constraints and sustain quality improvement in resource-limited settings [39].

### Potential interventions: training, feedback and workflow redesign

Clinicians strongly supported targeted interventions to address the identified challenges. Suggestions included structured induction and mandatory refresher training, provision of pre-prepared dual-set blood culture kits to reduce variability, and integration of prompts into sepsis pathways or electronic medical records. Importantly, participants emphasized the value of constructive, non-punitive feedback through visual dashboards reporting contamination rates, collection volumes and timeliness. These proposals are supported by evidence from metropolitan hospitals showing that such interventions can significantly reduce contamination and improve diagnostic yield [40,41]. However, participants noted that for feedback to be effective in rural contexts, data must be timely, locally relevant and presented in a way that fosters learning rather than blame.

### Indigenous health considerations

In two of the centres, participants noted additional challenges when caring for Aboriginal patients, including difficulties in venous access, language barriers in explaining the purpose of blood cultures and mistrust arising from previous negative healthcare experiences. These issues resonate with broader research documenting disparities in diagnostic access and outcomes for Aboriginal and other Indigenous populations [42,43]. Participants emphasized the need for culturally safe approaches, such as involving Aboriginal health workers to explain procedures and ensure respectful communication. Addressing these considerations is critical for equity and for ensuring that improvements in diagnostic practice benefit all patient groups.

### Integration with laboratory systems

A further theme concerned the disconnect between ED clinicians and regional laboratories. Participants described limited feedback on specimen quality, contamination or processing delays. Almost universally, clinicians had a poor understanding of how laboratories handle blood culture samples and felt that laboratories were ‘invisible’ partners in patient care. This lack of integration is consistent with studies showing that siloed laboratory-clinical communication contributes to missed learning opportunities [44,45]. A common theme was that once the laboratory processes were explained, the importance of sample quality became apparent. It is well recognized that knowledge of the right thing to do does not necessarily translate into practice unless operators understand the rationale. Clinicians recommended establishing two-way communication channels, such as regular feedback reports or joint quality-improvement initiatives, to strengthen collaboration and accountability.

### Implications for antimicrobial stewardship

These findings have important implications for antimicrobial stewardship in rural and remote health services. Missed or delayed blood cultures limit opportunities for pathogen-directed therapy and perpetuate reliance on empiric broad-spectrum antibiotics. This not only drives resistance but also exposes patients to unnecessary side effects and increases costs [46]. Conversely, contaminated cultures can trigger inappropriate antibiotic escalation, prolong hospitalization and undermine stewardship programmes [25]. Recalling patients after discharge based on a late ‘positive’ blood culture presents logistical problems in regional areas. Addressing barriers to blood culture quality is, therefore, a key strategy for improving antimicrobial use in sepsis care. Our findings suggest that stewardship interventions in rural EDs must go beyond prescribing audits to include diagnostic practices and feedback loops.

### Development of blood culture guidelines

As well as the technical aspects of blood culture collection, clinicians would welcome guidance on deciding which patients to investigate. More rational use of blood cultures would avoid unnecessary investigations of low-risk patients, thereby reducing the risk of false positives. Focusing instead on a smaller proportion of presentations where BSI is more likely to occur would assist compliance with best-practice sample collection.

### Strengths and limitations

This study provides novel insights into the lived experiences of frontline clinicians in regional Western Australia, adding depth to existing quantitative data on sepsis management. By capturing perspectives across multiple sites, the findings reflect a range of contextual challenges and enablers relevant to rural and remote health services. However, the study also has limitations. As with all qualitative research, findings are not statistically generalizable, though they are transferable to similar contexts. Participants were self-selecting, which may have introduced bias towards those more engaged with sepsis care. Finally, while interviews captured a breadth of views, observations of practice were not undertaken and reported behaviours may differ from actual behaviours.

## Conclusion

Overall, the study demonstrates that blood culture practice in rural EDs is shaped by systemic, workforce and cultural factors that extend beyond individual clinician behaviour. Improving diagnostic yield and reducing contamination requires a multi-level approach that addresses laboratory turnaround times, workflow design, staff training and feedback systems. Local leadership and team culture are key enablers, and targeted interventions can build on these strengths to support antimicrobial stewardship, efficient practice and enhance patient safety in resource-limited settings.

## Abbreviations

ADEPT: Adaptive Diagnostics for Emerging Pathogenic Threat
ED: emergency department
WACHS: Western Australia Country Health Service
